# PReS13-SPK-1572: The therapy management of pain amplification syndrome

**DOI:** 10.1186/1546-0096-11-S2-I36

**Published:** 2013-12-05

**Authors:** S Maillard

**Affiliations:** 1 Great Ormond Street Hospital, London, UK

## Objectives

1. Discuss presentation and assessment of pain syndromes, including CRPS

2. Present the bio - psychosocial model of management for these complex patients

3. Provide a review of different approaches to the therapy management

Pain syndromes often provide the clinician with the most complex challenges in understanding the condition as well as working out the maintaining factors and therefore how to support these patients back to function and recovery. These young people are often very disabled by these conditions and struggle to function in all aspects of their life including school and socially. The bio-psychosocial model of management involving all aspects of their life is the most successful and this often requires an effective multidisciplinary team in order for the outcome to be effective. The most important aspects of treatment are for the clinicians to be clear about the diagnosis and to stop investigations looking for a cause. Secondly the child and family need to realise that the most effective approach is based on 'active participation' in all aspects of the therapy with the goal being self-management and participation in all aspects of life fully.

Treatment should focus upon the young person completing exercises in order to regain movement, strength and stamina resulting in increased function. Work with desensitisation is also important, but not alone from regaining movement and strength. Learning specific pain management techniques, active relaxation and how to pace activities is also important. It is important to focus on solutions, recovery and independent self -management rather than causes as this can prevent recovery. Reintegration into normal activity is important and school attendance should be prioritised followed by returning to sport and other physical activities. The outcomes of effective management is very positive in young people providing they engage with the understanding that they are the solution to the management of the pain.

## Disclosure of interest

None declared.

